# Impact of *Neospora caninum* Infection on the Bioenergetics and Transcriptome of Cerebrovascular Endothelial Cells

**DOI:** 10.3390/pathogens9090710

**Published:** 2020-08-28

**Authors:** Hany M. Elsheikha, Mamdowh Alkurashi, Suzy Palfreman, Marcos Castellanos, Kenny Kong, Evita Ning, Nashwa A. Elsaied, Kalotina Geraki, William MacNaughtan

**Affiliations:** 1 Faculty of Medicine and Health Sciences, School of Veterinary Medicine and Science, University of Nottingham, Sutton Bonington Campus, Leicestershire LE12 5RD, UK; kurashi@ksu.edu.sa (M.A.); ntxsp16@exmail.nottingham.ac.uk (S.P.); nashw2@yahoo.com (N.A.E.); 2 Animal Production Department, College of Food and Agricultural Sciences, King Saud University, Riyadh 11451, Saudi Arabia; 3 Nottingham Arabidopsis Stock Centre, Division of Plant and Crop Sciences, School of Biosciences, University of Nottingham, Leicestershire LE12 5RD, UK; sbzmc3@exmail.nottingham.ac.uk; 4 School of Physics and Astronomy, University Park, University of Nottingham, Nottingham NG7 2RD, UK; kenny.kong@facemodata.co.uk; 5 Centre for Molecular Nanometrology, Department of Pure and Applied Chemistry, Technology and Innovation Centre, University of Strathclyde, Glasgow G1 1RD, UK; evita.ning@strath.ac.uk; 6 Diamond Light Source, Harwell, Didcot OX11 0DE, UK; tina.geraki@diamond.ac.uk; 7 Division of Food, Nutrition and Dietetics, School of Biosciences, University of Nottingham, Sutton Bonington Campus, Loughborough LE12 5RD, UK; sczbim@exmail.nottingham.ac.uk

**Keywords:** *Neospora caninum*, host–pathogen interaction, blood-brain barrier, infrared spectroscopy, Synchrotron-based XRF mapping, differential gene expression

## Abstract

In this work, the effects of the protozoan *Neospora caninum* on the bioenergetics, chemical composition, and elemental content of human brain microvascular endothelial cells (hBMECs) were investigated. We showed that *N. caninum* can impair cell mitochondrial (Mt) function and causes an arrest in host cell cycling at S and G2 phases. These adverse effects were also associated with altered expression of genes involved in Mt energy metabolism, suggesting Mt dysfunction caused by *N. caninum* infection. Fourier Transform Infrared (FTIR) spectroscopy analysis of hBMECs revealed alterations in the FTIR bands as a function of infection, where infected cells showed alterations in the absorption bands of lipid (2924 cm^−1^), amide I protein (1649 cm^−1^), amide II protein (1537 cm^−1^), nucleic acids and carbohydrates (1092 cm^−1^, 1047 cm^−1^, and 939 cm^−1^). By using quantitative synchrotron radiation X-ray fluorescence (μSR-XRF) imaging and quantification of the trace elements Zn, Cu and Fe, we detected an increase in the levels of Zn and Cu from 3 to 24 h post infection (hpi) in infected cells compared to control cells, but there were no changes in the level of Fe. We also used Affymetrix array technology to investigate the global alteration in gene expression of hBMECs and rat brain microvascular endothelial cells (rBMVECs) in response to *N. caninum* infection at 24 hpi. The result of transcriptome profiling identified differentially expressed genes involved mainly in immune response, lipid metabolism and apoptosis. These data further our understanding of the molecular events that shape the interaction between *N. caninum* and blood-brain-barrier endothelial cells.

## 1. Introduction

*Neospora caninum* is an obligate intracellular apicomplexan protozoan parasite. An association of this parasite with abortion in cattle [1,2] and neuromuscular disease in dogs has been established based on seroepidemiological and pathological studies [2,3,4,5,6]. Treatment and control of the disease caused by *N. caninum* remains problematic; due to the high costs [7], the difficulty in treating a eukaryotic pathogen in a eukaryotic host and the incomplete understanding of the host–parasite interaction, especially the process by which *N. caninum* penetrates the blood brain barrier (BBB) and causes neuropathies [8]. Although crossing the BBB is one of the hallmark features of *N. caninum* infection, the mechanisms underpinning this event and the progression to brain damage are not well understood.

The BBB is highly vulnerable to injuries due to parasite invasion and traversal to the brain. *N. caninum* must possess mechanisms that enable traversal of this complex interface, which separates the central nervous system (CNS) from the main blood supply [9]. The BBB is an active tissue made up of astrocytes, pericytes and microvascular endothelial cells, and selectively controls intracellular and paracellular passage of substances between the CNS and blood [9,10]. These cellular components along with closely packed neurons constitute the neurovascular component, which forms the major functional unit of the BBB [9]. Understanding the molecular changes that occur in the microvascular endothelial cells due to *N. caninum* infection is therefore important because these cells maintain the functional integrity of the BBB and provide a highly selective barrier that protects the brain against pathogen invasion. Earlier studies have revealed disruption of the bioenergetics of microvascular endothelial cells in response to *N. caninum* infection [11,12].

Transcriptomics analysis using microarray technology has been used to profile gene expression of host cells infected by *N. caninum* [13], yet this approach has not been applied in the context of BBB endothelial cell infection. The use of microarray analysis where mRNA transcription patterns of many thousands of genes are simultaneously revealed can identify previously unknown host factors and pathways that modulate host–parasite interaction. Likewise, the use of spectroscopic techniques, such as Fourier Transform Infrared (FTIR), can reveal alterations in the chemical constituents of infected cells [14]. Additionally, synchrotron radiation X-ray fluorescence (μSR-XRF) is a chemical element imaging technique that can be used to generate X-ray fluorescence elemental maps of biological tissues [15,16,17], with detection sensitivity and spatial resolution well-suited to characterize host–parasite interactions. Together, transcriptomics, spectroscopic FTIR and μSR-XRF approaches can provide an integrated global view of the transcriptional, chemical, and elemental changes that occur in BBB endothelial cells in response to *N. caninum* infection.

In this study, we used the FTIR method to identify chemical changes that occur in human brain microvascular endothelial cells (hBMECs) following *N. caninum* infection. The levels of the trace elements Zn, Fe, and Cu in the infected and control cells were determined using quantitative μSR-XRF imaging analysis. We also profiled the expression of 84 genes involved in the biogenesis and function of mitochondria using RT-PCR-based focused pathway analysis. Furthermore, we analyzed the global differences in the patterns of gene expression in human and rat brain microvascular endothelial cell lines following *N. caninum* infection using genomic cDNA microarrays. FTIR imaging of infected hBMECs revealed significant changes in the major chemical macromolecules of the infected cells. Gene expression profiling of infected human and rat microvascular endothelial cells has identified differentially expressed genes and biological processes that mediate the parasite survival and replication within the host cells.

## 2. Results

### 2.1. Effect of N. caninum on Cell Viability, Mt Function and Cell Cycle

**Figure 1:**
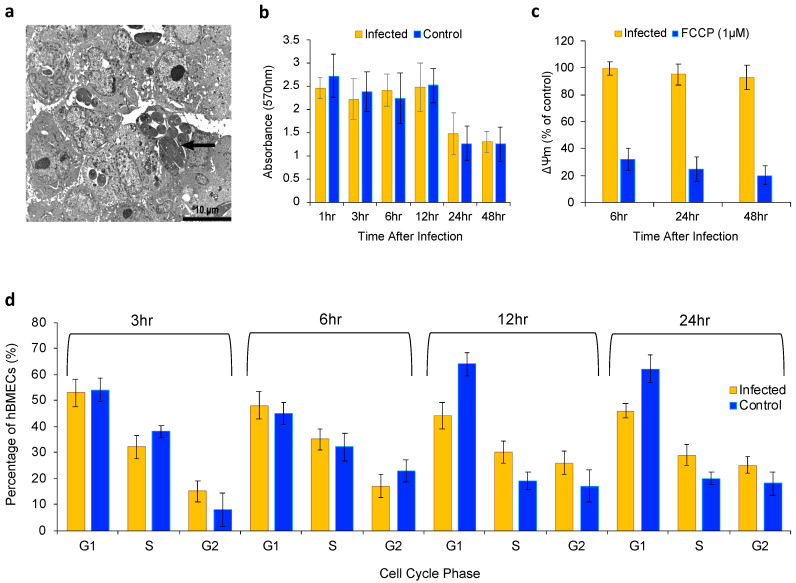
Effects of *Neospora caninum* on the viability, Mt function, and cycling of hBMECs. (**a**) TEM micrograph of *N. caninum*-infected hBMECs. The arrow points at several tachyzoites within the PV. (**b**) At 1, 3, 6, 12, 24, and 48 hpi, cell proliferation was measured using the (3-(4,5-Dimethylthiazol-2-yl)-2,5-diphenyltetrazolium (MTT) assay. Absorbance values did not differ significantly between infected and uninfected cells at any of the examined time points. (**c**) At 6, 24, and 48 hpi the ΔΨm of hBMECs was measured using MitoTracker^®^ Green FM. A modest reduction in ΔΨm was observed at 24 and 48 hpi in infected cells compared to the uninfected controls. (**d**) The proportion of the G1, S, and G2 phases were measured in *N. caninum*-infected or non-infected hBMECs. At the indicated times, cells were harvested, and the cell cycle profile was determined using propidium iodide staining and flow cytometry. *N. caninum* infection caused transient cell cycle arrest in S and G2 phases. All results represent the means ± standard deviations (SD) of three independent experiments, each was performed in quadruplicate.

The presence of *N. caninum* tachyzoites within the parasitophorous vacuole (PV) inside hBMECs was confirmed by TEM analysis (Figure 1a). There was no significant difference in cell viability between infected and non-infected hBMECs at any time point after infection (Figure 1b); however, there was a modest reduction (*p* > 0.05; two-way ANOVA) in the mitochondrial membrane potential (ΔΨm) in infected hBMECs at 24 and 48 h post-infection (hpi) (Figure 1c). We observed an increase in the proportions of *N. caninum*-infected cells in S and G2 phases of the cell cycle at 12 and 24 hpi (Figure 1d), indicating that *N. caninum* tachyzoites have slightly reduced the proliferation of infected cells compared to uninfected cells (*p* > 0.05; two-way ANOVA). Next, we used RT-PCR to analyze the expression of a focused panel (RT^2^ Profiler™ PCR Array Kit) of 84 genes involved in the host cell Mt respiration, including genes encoding components of the electron transport chain and oxidative phosphorylation (OXPHOS) complexes. Among the analyzed 84 Mt genes, the expression of 21 genes was significantly increased (*p* < 0.05 and fold change (FC) >1.5) and 3 of these genes showed a greater than 2 fold increase in infected hBMECs compared to the control cells (Table 1). These included *ndufc2* (NADH dehydrogenase (ubiquinone) 1, subcomplex unknown, 2, 14.5kDa; FC 2.37), *cyc1* (Cytochrome c-1; FC 2.37), and *atp4a* (ATPase, H+/K+ exchanging, alpha polypeptide; FC 2.32). These results indicate that an increase in host cell bioenergetics can accompany *N. caninum* infection.

### 2.2. FT-IR Spectral Analysis

**Figure 2:**
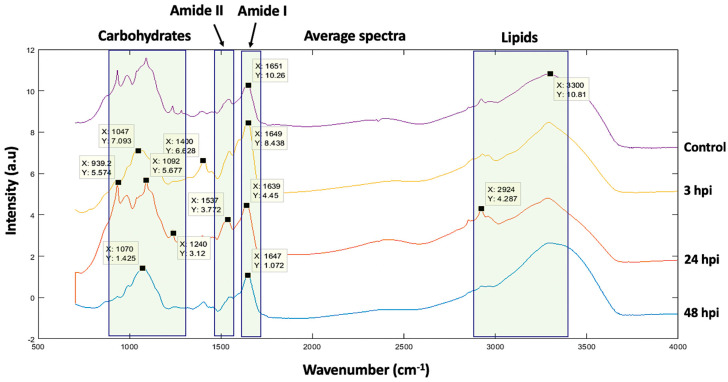
Time-resolved average IR spectra of human cerebrovascular endothelial cells at 3, 24 and 48 hpi by *Neospora caninum* compared to uninfected (control) cells. The positions of the major IR bands are indicated. Most of the significant differences lie in the fingerprint (900–1800 cm^−1^) region and high wavenumber region (2900–3300 cm^−1^). Spectra are *y*-axis shifted for clarity.

As shown in Figure 2, IR analysis of hBMECs assigned the major bands in vibrational spectra of control and infected hBMECs at different time points after infection, to absorption bands of lipid, protein (amide I, amide II), carbohydrates and nucleic acids, showing peaks at 2924 cm^−1^ (lipid), 1649 cm^−1^ (amide I protein), 1537 cm^−1^ (amide II protein) and 1092 cm^−1^, 1047 cm^−1^, and 939 cm^−1^ (nucleic acids and carbohydrates).

**Figure 3:**
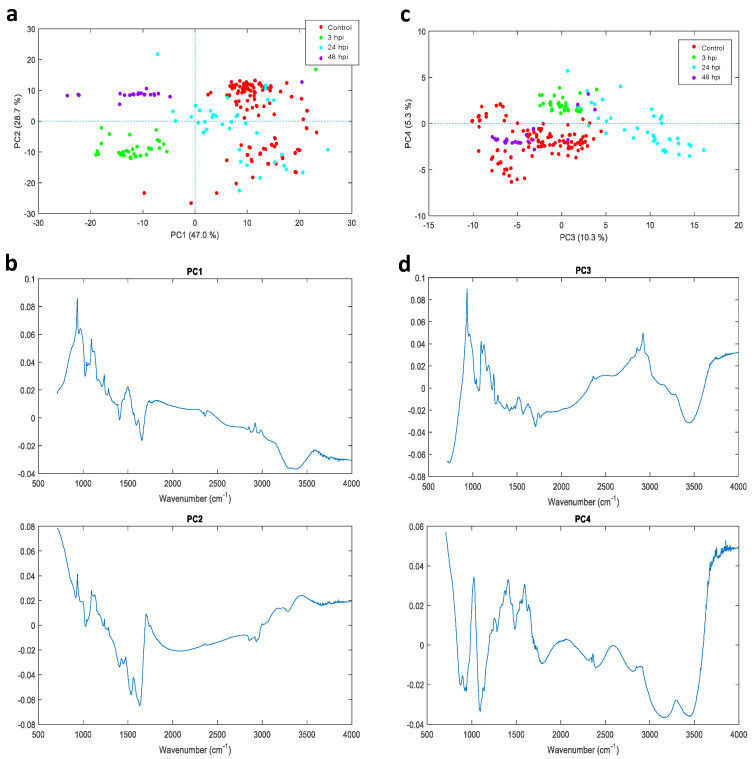
Principal components analysis (PCA)-based discrimination of IR patterns of *Neospora caninum*-infected and uninfected hBMECs with respect to time post infection. (**a**) PCA score plots of PC1 versus PC2 for intracellular FTIR chemical fingerprints of infected versus uninfected (control) cells. PC1 and PC2 explained 47% and 28.7% of the variance in the IR spectral data, respectively. Samples at different time points are indicated by different colors. The spectra for 24 hpi and control samples were not clearly separated. However, PC1 shows clear spectral differences between the 2 groups (3 hpi and 48 hpi) and (control and 24 hpi). PC2 shows a clear separation between 3 hpi and 48 hpi. (**b**) Loading plots for the PCAs 1 and 2 shown in (**a**). For PC1 the positive bands located at 939 cm^−1^ and 1092 cm^−1^ are the most significant features that distinguish control and 24 hpi samples. For PC2, the negative 1649 cm^−^^1^ band represents the most significant feature that distinguishes between the 3 hpi and 48 hpi samples. (**c**) PC3 had the most discriminatory power for differentiating between control and 24 hpi samples. (**d**) The loading plots show that the positive band in the region of 1000 cm^−1^ that appears in PC3 is a strong feature for distinguishing the control from 24 hpi, whereas for PC1 this feature was relatively less effective in distinguishing the control from the 24 hpi samples due to contributions from other bands.

The PCA (principal components analysis) score plots PC1 and PC2, provided the most discriminating power for differentiation of samples (Figure 3a,b). The spectra of 24 hpi and control samples were not clearly separated. However, PC1 showed the most the discriminating power to separate the 2 groups of samples (3 and 48 hpi) and (control and 24 hpi). The positive bands at 939 cm^−1^ and 1092 cm^−1^ were most significant in the discrimination between these 2 groups. The negative band at 1649 cm^−1^ was also significant in distinguishing the 3 hpi and 48 hpi samples from the control and 24 hpi group. PC2 discriminated between 3 hpi and 48 hpi samples. The negative 1649 cm^−1^ band in PC2 was the most significant band separating the 3 and 48 hpi samples. PC1 and PC2 were unable to discriminate between all samples (i.e., control, 3, 24, and 48 hpi). The control and 24 hpi could be separated by the 2924 cm^−1^ band as well as other bands in the carbohydrate region in PC3 (Figure 3c,d) in PC3. The intensity of the 2924 cm^−1^ band was greater in the 24 hpi and lower in the control spectra. The average spectra for control cells and cells infected at 3, 24 and 48 hpi are shown in Supplementary Figure S1. These results show that hBMECs exhibit distinct chemical signatures following *N. caninum* infection and that FTIR with PCA can reveal the chemical differences in cells at different time points after infection, and between infected and control cells.

### 2.3. XRF Elemental Mapping

**Figure 4:**
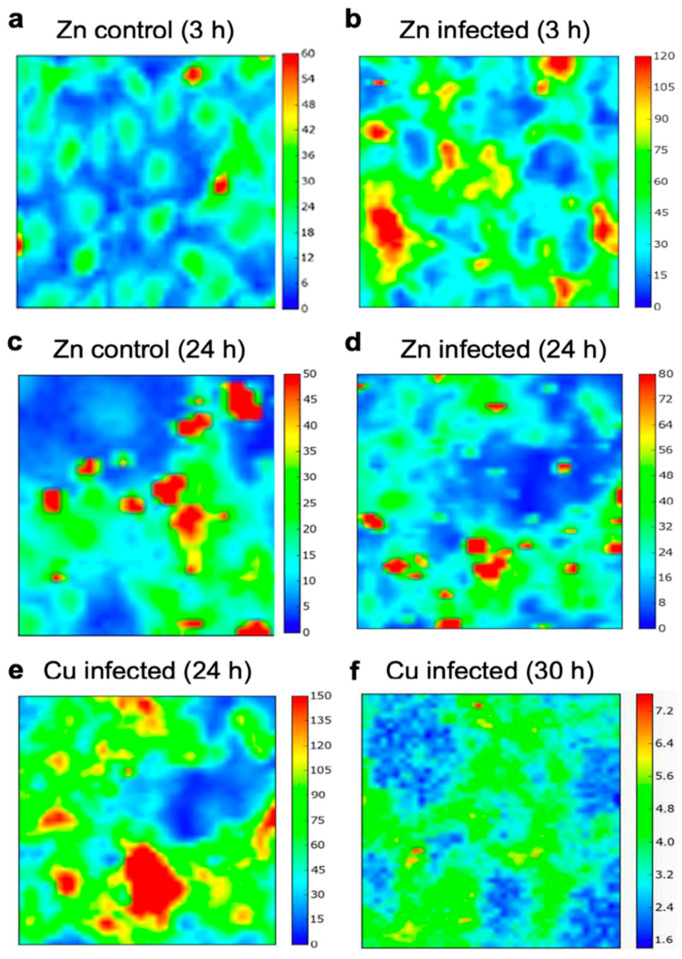
Representative elemental maps for the high-resolution μSR-XRF analysis. (**a**–**d**) Zinc (Zn) profile in hBMECs infected by *Neospora caninum* at (**b**) 3 and (**d**) 24 hpi compared to control cells (**a**–**c**) at the same incubation periods post infection. (**e**,**f**) Cupper (Cu) distribution in *N. caninum*-infected hBMECs showing significant drop in Cu from 24 h (**e**) to 30 h (**f**) after infection. The rainbow-colored scale bar gives the signal intensity and corresponding elemental concentration in the range presented for each map.

As trace metals can be associated with different phases of infection, we hypothesized that alterations in the elemental content of host cells during the intracellular parasite development are significant and can be observed by spatially resolving and averaging the cellular levels of key trace elements. Elemental maps of *N. caninum*-infected and uninfected hBMECs were obtained using μSR-XRF, which gave novel insights into the influence of *N. caninum* on the hemostasis of 3 major trace elements (Zn, Cu and Fe) inside host cells at 3, 24 and 30 hpi. The elemental concentration and spatial distribution in the hBMEC monolayer changed markedly with regard to different trace elements and as *N. caninum* infection progressed within host cells. Figure 4 shows representative images of the distribution of Zn, Cu and Fe. Altered concentrations of Zn, Cu and Fe were identified using a synchrotron microprobe at 3 µm spatial resolution. The overall statistical comparison of the metal levels in infected cells suggest an increase in Zn (*p* < 0.05) when compared to the control cells of equivalent incubation time, while for Fe and Cu the variation from the controls does not appear to be significant (*p* < 0.05). Amongst the different incubation times of the infected cells, the levels of both Zn and Cu increased from 3 hpi (17.66 ± 1.65 for Zn and 7.98 ± 2.36 for Cu) to 24 hpi (20.58 ± 2.01 for Zn and 28.97 ± 1.31 for Cu), whilst between 24 and 30 hpi there was a large drop of Cu from 28.97 ± 1.31 to 3.22 ± 0.46 (*p* < 0.05). Fe did not vary significantly with the incubation times, suggesting that Fe might not be involved in the host–parasite interaction (*p* > 0.05).

### 2.4. N. caninum Infection Induced Modest Transcriptional Changes

We examined the global gene expression changes in hBMECs or rBMVECs exposed to *N. caninum* tachyzoites for 24 h using Affymetrix GeneChip human gene 1.0 ST array or Rat Genome 230 2.0 array, respectively. Gene expression data of *N. caninum*-infected human or rat cell line were compared to the expression profile of the mock-infected cells to determine the relative modulation of cellular transcription induced by infection. The numbers of differentially expressed genes (DEGs) in comparison with uninfected (control) cells were seven for hBMECs and 10 for rBMVECs, indicating that *N. caninum* infection altered the expression of only 0.024% of human genes and 0.047% of rat genes. Expression of genes was higher in rBMVECs relative to hBMECs, suggesting the presence of host species-specific differences in the biological response to *N. caninum* infection. From the seven DEGs in hBMECs, two were upregulated, and out of 10 DEGs in rBMVECs, only one gene (*Gem*) was upregulated, and nine were downregulated. As shown in Table 2, the top seven DEGs in infected hBMECs included the following five downregulated genes: NADH dehydrogenase 2 (*mtnd2*; FC −4.143), insulin induced gene 1 (*insig1*; FC −1.705), histone cluster 1, H2ak (*hist1h2ak*; FC–1.678), 3-hydroxy-3-methylglutaryl-CoA synthase 1 (*hmgcs1*; FC −1.636), and small nucleolar RNA, H/ACA box 7B (*snora7b*; FC −1.600), and two upregulated genes: transducer of ERBB2, 2 (*tob2*; FC 1.860) and basic, immunoglobulin-like variable motif containing (*bivm*; FC 1.520).

For infected rBMVECs, there was one upregulated gene GTP binding protein overexpressed in skeletal muscle (*gem*; FC 1.555) and nine downregulated genes, including radical S-adenosyl methionine domain containing 2 (*rsad2*; FC −2.046), chemokine (C-X-C motif) ligand 2 (*cxcl2*; FC −2.040), tribbles homolog 3 (Drosophila) (*Trib3*; FC −1.818), midasin homolog (yeast) (*mdn1*; FC −1.626), interferon, alpha-inducible protein 27 like 2B (*ifi27l2b*; FC −1.565), chemokine (C-X-C motif) ligand 10 (*cxcl10*; FC −1.544), HECT and RLD domain containing E3 ubiquitin protein ligase family member 1 (*herc1*; FC −1.543), growth arrest and DNA-damage-inducible, alpha (*gadd45a*; FC −1.528), and ISG15 ubiquitin-like modifier (*Isg15*; FC −1.508). The majority of the DEGs are involved mainly in immune response and lipid biosynthetic processes.

### 2.5. Clustering and Gene Ontology Enrichment Analysis of DEGs

**Figure 5:**
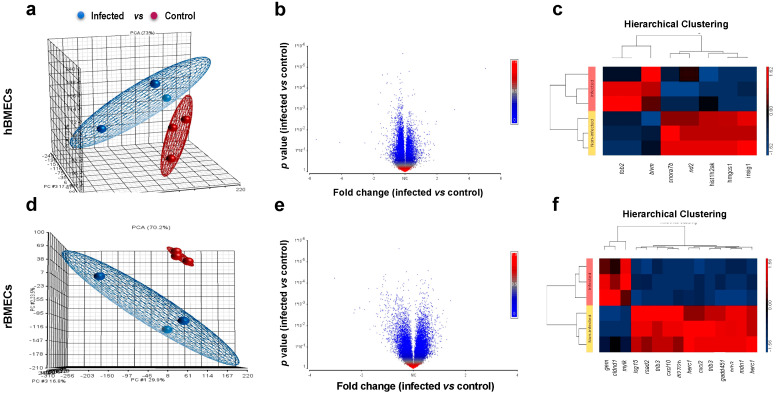
The effects of *Neospora caninum* on the expression levels of the genes of hBMECs and rBMVECs determined by microarray analysis at 24 hpi. PCA scatter plots of gene expression profiles of (**a**) hBMECs and (**d**) rBMVECs showing a clear infection-dependent effect in both cell lines. The plots demonstrate a clear separation in the expression profile of infected and uninfected (control) cells. (**b**,**e**) Volcano plots showing individual genes as dots. The red and blue dots represent genes with increased and decreased expression, respectively. Genes whose expression levels were not significantly changed are shown in grey. *X*-axis shows Fold Change (FC) and *y*-axis shows *p*-value. (*n* = 3/group; *p*-value < 0.05; FC ± 1). (**c**,**f**) Heat map showing hierarchical clustering of DEGs between infected and uninfected hBMECs and rBMVECs at 24 hpi. Each group had three replicates. Red and blue colors indicate genes with increased and decreased expression, respectively. Gene expression in infected cells was compared to uninfected cells and the criterion for differential expression was ≥ 1FC with a *p*-value of < 0.05.

PCA was performed on the whole gene set. As shown in Figure 5a,d, PCA identified spatial separation as a function of infection for both hBMECs and rBMVECs and their controls, supporting species-specific and infection-specific clustering. Volcano plots were also used to show individual genes plotted as dots that were either relatively enriched or suppressed (Figure 5b,e). Also, the identified DEGs were processed for hierarchical clustering, which showed a close assembly of gene expression profiles separating infected from control cells (Figure 5c,f). We also performed gene ontology (GO) enrichment analysis of the DEGs of hBMECs to gain insight into the biological processes regulated during *N. caninum* infection. There were 32 biological process GO terms, and the top 10 most significantly enriched GO terms were cholesterol biosynthetic process, response to sterol depletion, response to purine-containing compound, negative regulation of steroid biosynthetic process, response to gonadotropin stimulus, cellular response to cholesterol, cranial suture morphogenesis, response to acid, cellular response to follicle-stimulating hormone stimulus, and negative regulation of fatty acid biosynthetic process. For the molecular function category, seven GO terms were enriched, including vitamin D receptor binding, drug binding, isomerase activity, catalytic activity, protein homodimerization activity, transferase activity, and protein binding. In the cellular component category, eight GO terms were detected, including cytoplasm, soluble fraction, endoplasmic reticulum membrane, endoplasmic reticulum, nucleus, cytosol, membrane, and integral to membrane.

## 3. Discussion

In this study, we used a combination of approaches to investigate the effects of *N. caninum* infection on the bioenergetic, chemical constituent, elemental content, gene expression, and biological processes of brain microvascular endothelial cells. Our results showed that *N. caninum* tachyzoites were able to invade and replicate within hBMECs without significantly altering the cell viability or mitochondrial (Mt) function up to 48 hpi. This is consistent with a previous study, which showed that *N. caninum* can infect hBMECs without disrupting the cell viability or Mt integrity [11]. Mt gene expression profiling detected increased expression of 21 genes out of the 84 Mt examined genes. These upregulated Mt genes contribute to the function of Complex I (NADH-Coenzyme Q Reductase), Complex II (Succinate-Coenzyme Q Reductase), Complex III (Coenzyme Q-Cytochrome c Reductase), Complex IV (Cytochrome c Oxidase), and Complex V (ATP Synthase). For example, *cyc1* gene (cytochrome c1) encodes a subunit of the cytochrome bc1 complex, which plays an important role in the Mt respiratory chain by transferring electrons from the Rieske iron-sulfur protein to cytochrome c. Mutations in the *cyc1* gene may cause Mt complex III deficiency, nuclear type 6. Mt staining in a previous study [11] and the present study did not reveal significant changes in the host cell Δψm up to 48 hpi. However, increased oxygen consumption was observed previously in *N. caninum*-infected hBMECs [11] and *N. caninum*-induced enhanced expression of Mt genes was detected in the present study (Table 1). These results suggest a modest increase in host cell bioenergetics to mitigate the increased metabolic pressure caused by the growing parasites within the host cells. Besides the basic function of providing energy, Mt are involved in various biological processes, such as calcium homeostasis, apoptosis, and cellular defense mechanisms. Hence, it is reasonable to assume that *N. caninum* tachyzoites target and alter some of the Mt functions to promote their own survival.

Infection of hBMECs by *N. caninum* was associated with a cell cycle arrest at the S and G2 phases. G2 phase is a period of rapid cell growth and protein synthesis during which the cells prepare for mitosis. Therefore, it is sensible to hypothesize that *N. caninum* induces cell cycle arrest at G2/M phase, without compromising the overall viability of infected host cells, to promote favorable conditions for sustaining the parasite proliferation. *N. caninum* has been previously thought to interfere with the host cell cycle or apoptotic pathways to promote its own intracellular survival [11,18]. Many pathogens, including *N. caninum*, have evolved strategies to interrupt apoptosis to keep host cells alive until the pathogen development is completed. *N. caninum* infection can inhibit apoptosis of mouse embryonic fibroblasts by inhibiting caspase activity [19]. The same process may be occurring during *N. caninum* infection of hBMECs. The closely related apicomplexan protozoan *Toxoplasma gondii* is also known to alter the host cell cycle and apoptotic pathway [20,21,22]. On the contrary, NcROP16, a key virulence factor in *N. caninum*, can promote host cell apoptosis via increasing the transcription of apoptotic-related genes, such as *Fas*, *FasL* and *Bax*, while enhancing the parasite pathogenicity by phosphorylating STAT3 [23]. Given the distinct roles of *N. caninum* in cell cycling and apoptotic pathways of host cells, future studies should characterize the molecular mechanisms underlying *N. caninum*-induced cellular arrest and reveal host cell targets of *N. caninum*-related proapoptotic effector proteins.

FTIR spectroscopy was used to the study response of hBMECs to *N. caninum* infection at 3, 24 and 48 hpi by determining the chemical changes that occur in the host cells as infection advances. The detected chemical changes were significant at early infection (3 hpi) and late infection (48 hpi), prior to cell damage caused by the parasites exiting host cells. The FTIR profile seems to correlate with the progression of the parasite infection cycle. For example, during early infection (3 hpi) tachyzoites invade and establish a PV within host cells. At late infection (48 hpi), the tachyzoites have completed their development and are ready to exit the cells by rupturing the cell membrane. Both time points are associated with invasive events and more alterations are expected to happen at these two time points. However, at 24 hpi we noticed that the host cells’ metabolic status became more stabilized, probably to allow the tachyzoites to develop within the PV. We are cognizant that these chemical changes could be a direct effect of the cellular response to infection or an added parasite metabolome to the host cell metabolome. Chemometric analysis showed that PC1, PC2 and PC3 were required to discriminate between all samples. Alteration of the main chemical constituents of the host cells is anticipated because *N. caninum* is an intracellular pathogen and most of its energy and metabolic requirements, which are crucial for its growth and survival, are derived from the host cells [11,12]. Previous work has also demonstrated high expression of genes involved in the growth of the virulent *N. caninum* isolate Nc-Spain7, including genes involved in DNA replication, RNA metabolism, protein synthesis, cell division, and energy production [24].

μSR-XRF mapping of *N. caninum*-infected hBMECs revealed, for the first time, alterations in the levels of trace elements Zn and Cu in response to *N. caninum* infection. Zn, Cu and Fe are essential trace elements required for a multitude of cellular housekeeping functions, such as enzymatic reactions, DNA synthesis, metabolic processes, and gene expression [25]. Hence, homeostasis of these elements within cells is tightly regulated, and alteration in their levels can have an adverse impact on the host cell and its ability to respond to microbial infection. Of special interest is the remarkable increase in Zn in infected cells compared to control cells. The increase in Zn as infection progressed within cells suggests the important role of Zn in host immune defense against this intracellular parasite. *N. caninum* infection induced the production of Th1-immune cytokines, (e.g., interleukin-12 (IL-12), interferon gamma (IFN-γ)) and increased the levels of reactive oxygen species [4]. Also, *N. caninum* reduced host cell apoptosis by inhibiting caspase activity [19]. Zinc deficiency in mammals can adversely affect Th1 type immune response and IL-2 production [26,27] and apoptosis [28]. Therefore, a correlation exists between increased Zn level and immune response against *N. caninum*, and the ability of *N. caninum* to impair host cell apoptosis; mechanisms that are critical for parasite survival within host cells. Hence, it is possible that increased Zn level—whether as a host cell response or parasite-induced—has a role to play in the protection against the cellular oxidative stress and DNA damage [29,30] produced during infection, suppression of cell apoptosis [28], and enhancing cell mediated immunity [26,27]. The availability and restriction of Fe and Cu are also important aspects of host–pathogen interactions [31]. However, the low level of Fe in the present study raises the possibility that this element might not be essential for *N. caninum* intracellular growth. On the other hand, the rise of Cu from 3 to 24 hpi, followed by a sharp decrease at 30 hpi appears advantageous to the host–parasite interplay because excess Cu is toxic to the host cell and catalyzes the generation of free oxygen radicals that may damage host cell lipids, proteins and DNA, as well as the parasite [32]. Hence, this particular trend of Cu levels might be part of the cellular response and the homeostatic control of Cu by host cells during infection.

We used genomic cDNA microarrays to profile the transcriptional changes that occur in hBMECs and rat rBMVECs, the first cell type thought to be targeted during cerebral *N. caninum* infection. The ability to compare and contrast gene expression profiles between endothelial cells of human and murine origins provided new insights into the differences between human and murine response to *N. caninum* infection. PCA of the full data set showed distinct separation between infected and control cells for both hBMECs and rBMVECs. Likewise, hierarchical clustering analysis using heat maps revealed a clear separation between infected and control cells in both cell lines, demonstrating *N. caninum*-dependent effect on host cell transcriptional response. Moreover, it was striking to see such a modest effect of *N. caninum* infection on gene expression of host cells, which indicates that endothelial cell transcriptional responses to *N. caninum* 24 hpi are limited, or it could be an evasion strategy by the parasite to subvert the host immune responses. Indeed, microarray data and the functional annotation of DEGs clearly showed that *N. caninum* can adapt well to the host cell microenvironment by inducing genes and processes that promote the intracellular parasite growth. These data are supported by the results of MTT assay, Mt gene expression, chemical fingerprinting and elemental analysis, which all-together showed how *N. caninum* effectively modulates the host cell microenvironment in various ways to sustain its survival.

The top five downregulated genes in hBMECs were *mtnd2*, *insig1*, *hist1h2ak*, *hmgcs1*, and *snora7b*. The *mtnd2* is a mitochondrial gene coding for the NADH dehydrogenase 2 (ND2) protein, which is a subunit of the mitochondrial membrane respiratory chain NADH dehydrogenase (Complex I). Its significant downregulation supports the concept of metabolic pressure associated with *N. caninum* infection. The *insig1* has been shown to play a role in innate immune response, which is essential for the host to counter infection [33]. The *hist1h2ak* encodes the nuclear histone H2A type 1 protein, which is responsible for the nucleosome structure of the chromosomal fiber, but its role in *N. caninum* infection is unknown. The *hmgcs1* encodes a protein in the mevalonate pathway, which provides molecules for cholesterol synthesis [34], and thus may play a role in lipid metabolism, which is required for the synthesis of the plasma membrane of the proliferating tachyzoites during the parasite’s asexual replication. The role of this gene in protozoal growth has been reported in a previous study, which showed that chemical inhibition of *hmgcs1* significantly inhibited the proliferation of the coccidian protozoan *Eimeria bovis* [35]. Hence, downregulation of *hmgcs1* might be a host cell strategy to limit the availability of essential nutrients for the parasite growth. Interestingly, chemical FTIR fingerprinting results showed that at 24 hpi, the cell chemical constituents were closer to control values than to early (3 hpi) and late (48 hpi) infections. Thus, it is possible that inhibition of *hmgcs1* is part of an overall calming-down event that occurs at 24 hpi. The role of the *snora7b* gene in *N. caninum* infection is unknown, but *snora/ds* seems to improve cellular resistance to multiple viruses and has been hypothesized to play a role in virus-host interactions and/or virus-induced cell death [36]. The top two upregulated genes in hBMECs were *tob2* and *bivm*. The *tob2* belongs to a family of antiproliferative proteins, which are involved in the regulation of cell cycle progression. The *bivm* encodes a poorly studied protein and its role in *N. caninum* is unknown.

For rBMVECs, there was one upregulated gene (*gem*) that is known to play a role in receptor-mediated signal transduction. There were nine downregulated genes, including radical S-adenosyl methionine domain containing 2 (*rsad2*), chemokine (C-X-C motif) ligand 2 (*cxcl2*), tribbles homolog 3 (Drosophila) (*Trib3*), midasin homolog (yeast) (*mdn1*), interferon, alpha-inducible protein 27 like 2B (*ifi27l2b*), chemokine (C-X-C motif) ligand 10 (*cxcl10*), HECT and RLD domain containing E3 ubiquitin protein ligase family member 1 (*herc1*), growth arrest and DNA-damage-inducible, alpha (*gadd45a*), and ISG15 ubiquitin-like modifier (*Isg15*). The *rsad2*, *cxcl2*, *ifi27l2b*, *cxcl10*, and *Isg15* play roles in immune response; *trib3* plays a role in fatty acid biosynthesis; *mdn1* is involved in transcription, *herc1* in membrane trafficking, and *gadd45a* in apoptosis.

Previous gene expression profiling of spleen cells in mice infected by *N. caninum* revealed differential expression of genes involved in cell proliferation, apoptosis, signal transduction, and transcription [13]. In the present study, changes in gene expression in infected hBMECs included genes involved in bioenergetics, lipid synthesis, cell cycling and immune response, whereas infected rBMVECs exhibited differential expression in genes involved in immune response, membrane trafficking, fatty acid biosynthesis, transcription, apoptosis and signaling. There was a similarity between hBMECs and rBMVECs in terms of involvement of DEGs in lipid biosynthesis. This is anticipated because *N. caninum* requires lipids to meet the high metabolic and biosynthetic demands of the growing and newly generated tachyzoites. Previous studies also showed that *N. caninum* relies on plasma lipoproteins, scavenges cholesterol from NPC1-containing endocytic organelles and salvages sphingolipids from host Golgi Rab14 vesicles that the parasite sequesters into its PV [37] and dysregulates mammalian lipid droplet biogenesis [38]. The *insig1* is known to block the transcription of SREBP-1c (sterol regulatory element binding protein-1c), which leads to the inhibition of lipid biosynthesis [39]. Thus, reduced expression of *insig1* is induced by *N. caninum* most likely to promote the production of lipid synthesis for its own benefits. Evidence from our and previous studies highlight lipid metabolism as a promising target for therapeutic interventions. The down regulation of genes involved in immune response in both hBMECs and rBMVECs is noteworthy. The immune response against *N. caninum* is predominantly Th1-biased, which is associated with the production of IL-12, IFN-γ, and nitric oxide (NO) by immune cells, such as macrophages, which underline the importance of cellular immune responses in the reduction of tissue parasitism and host survival, along with IgG2 production [40,41]. Previous studies demonstrated that initial *N. caninum* recognition includes Toll-like receptor 2 (TLR2), TLR3, and TLR11 [42,43]. Engagement of these receptors can trigger the activation of MyD88 or TRIF-dependent pathways, which induce the immune response against *N. caninum* [43,44]. CCR5 is also a key player in the immune response against *N. caninum* through the production of cyclophilin, a parasite protein that modulates migration and activation of innate immune cells during early infection [40,45].

The differences in gene expression and pathways observed in our study compared to previous work [13] are expected. It is possible that endothelial cells interact differently with *N. caninum* compared with other host cell types. *N. caninum* interaction with and crossing of BBB endothelial cells is a special event because BBB has highly selective properties to protect the CNS from pathogen invasion [46]. The strain of *N. caninum* (Nc-Liverpool) used in our study is also different from Nc-Nowra strain used by Ellis et al. [13]. Any comparison between in vitro and in vivo studies should be made with caution, as the results of in vivo studies may be influenced by the host’s biological processes, whereas many of these variables are absent in the in vitro environment. As our investigation had an exploratory nature, only genes present on the microarray were investigated, other genes may be of relevance to the outcome of *N. caninum* infection. The microarray analysis was conducted at only one time point in the human and rat cell lines. A time course study is required to reveal more temporal changes in gene expression during the full course of infection. Further studies should also compare host cell responses to infection by *N. caninum* strains of different virulence.

## 4. Materials and Methods

### 4.1. Cell Lines and Culture Conditions

Human brain microvascular endothelial cells (hBMECs) provided by Naveed Khan (American University of Sharjah, Sharjah, UAE) were grown in complete RPMI-1640 Medium (cRPMI) supplemented with 10% heat inactivated fetal bovine serum (FBS), 2 mM L-glutamine, 1 mM Sodium Pyruvate, 1% MEM nonessential amino acids, 1% MEM vitamins and 2% penicillin/streptomycin as previously described [11]. Rat Brain Microvascular Endothelial Cell line (rBMVEC), GPNT strain, was obtained from the European Collection of Authenticated Cell Cultures (ECACC, Health Protection Agency Culture Collections (HPACC), Salisbury, UK). Commercially prepared RBMVEC growth medium (ECACC, HPACC, Salisbury, UK) was used to maintain the rBMVEC culture. The rBMVECs were cultured in monolayer in flasks previously coated with Attachment Factor Solution (AFS), following the manufacturer’s instructions. African Green Monkey (*Cercopithecus aethiops*) kidney epithelial (Vero) cells were obtained (ECACC, HPACC, Salisbury, UK) and grown in Dulbecco’s Modified Eagle Medium (DMEM) supplemented with 10% heat-inactivated fetal bovine serum, 25 mM HEPES, 2 mM L-glutamine, 10,000 U/mL penicillin G sodium, 10,000 μg/mL dihydrostreptomycin, 250 μg/mL amphotericin B, nonessential amino acids, and 100 mM sodium pyruvate. Vero cells were used to maintain the growth of *N. caninum* tachyzoites.

All cell lines were maintained in a monolayer in T-75 cm^2^ culture flasks in a humidified 5% CO_2_-95% air at 37 °C. Cells were considered confluent when their expansion had reached a point where cells touched each other on all sides and no intercellular gaps were observed. When cells reached at least 80% confluency (~3–5 days), cell monolayers were trypsinized using trypsin-EDTA (Thermo Fisher Scientific, Waltham, MA, USA) and cell suspension were seeded in new flasks at a 1:3 passage ratio for hBMECs and Vero cells. In rBMVECs, an aliquot of trypsinized cell suspension was used to determine the number of cells/mL using a hemocytometer, and new culture flasks/plates were inoculated at 6 × 10^3^ cells per cm^2^. To rule out if cell viability could be regarded as a factor affecting host cell–parasite interactions, which in turn might affect subsequent measurements, cell viability was assessed on a minimum of 100 cells using trypan blue exclusion assay prior to inoculation onto culture plates or flasks. Only cells with >98% viability were used.

### 4.2. Parasite Maintenance and Purification

Tachyzoites of *N. caninum* Nc-Liverpool strain were maintained in Vero cell monolayers grown in 75 cm^2^ culture flasks, as described previously [18]. The inoculated cell culture was maintained in 15 mL of complete DMEM at 37 °C, 5% CO_2_. The parasites were harvested when lysis of the Vero cells, due to infection, was completed, or until very few host cells remained intact, and a new Vero cell culture was inoculated in order to establish an on-going *N. caninum* culture. Once nearly all of the Vero cells had been lysed by the parasite, the culture flask was gently agitated, and the entire contents were transferred to a 50 mL falcon tube. The contents were left to settle for 10 min and any floating cell debris was removed by pipetting, being careful not to disturb the parasites towards the bottom of the solution. The tube containing the parasite was then centrifuged at ambient temperature at 2000× *g* for 5 min. The supernatant was carefully removed, and the parasite pellet re-suspended in 2.5 mL of sterile 1× Phosphate Buffered Saline (PBS; pH 7.2). The parasite suspension was then purified using PD-10 desalting columns filled with Sephadex-25 gel filtration material (GE Healthcare, Amersham, UK), as previously described [47]. The purified parasites were then centrifuged at 3000× *g* for 5 min. The supernatant was carefully removed, and the purified parasite pellet was re-suspended in 5 mL of the corresponding culture media (RBMVEC growth medium for rBMVECs and RPMI for hBMECs). An aliquot was used to count the number of tachyzoites per milliliter using a hemocytometer.

### 4.3. Transmission Electron Microscopy

hBMECs were cultured on poly-l-lysine-coated coverslips overnight prior to infection by tachyzoites of *N. caninum* at a host:parasite ratio of 1:2 (i.e., MOI; multiplicity of infection of 2). Twenty-four hours after incubation, cells were fixed in 1% glutardialdehyde-4% formaldehyde in 0.1M PBS at 4 °C for at least 2 h before processing. Samples were washed in 0.1 M PBS at 4 °C for 12 h, followed by a secondary fixation in 1% osmium tetraoxide-1.5% K_4_Fe(CN)_6_ in 0.1 M PBS (pH 7.2). Samples were washed twice in distilled water for 30 min and dehydrated in ethanol 50, 50, 70, 70, 90, 90, and 96% for 10 min and twice in ethanol 100% for 15 min. Samples were impregnated in equal volumes of epoxy resin (LX112) and ethanol 100% for 60 min at ambient temperature, followed by pure Epoxy resin (LX112) for 60 min at 37 °C. Resin was allowed to polymerize for 12 h at 60 °C. Sectioning of blocks was performed on a type LKB IV ultramicrotome at 40 nm, and then the sections were collected on a copper 200-mesh grid. Sections were incubated with 6% uranyl acetate for 10 min, followed by lead citrate for 1 min, before they were viewed under a Philips Morgangi 268 transmission electron microscope connected to a charge-coupled device camera (MegaView II).

### 4.4. Cell Viability Assessment Using MTT Assay

The nonradioactive colorimetric 3-(4,5-dimethyl-2-thiazolyl)-2,5- diphenyl tetrazolium bromide (MTT, Sigma) reduction assay was used to determine the effect of *N. caninum* infection on the viability of hBMECs at 1, 3, 6, 12, 24, and 48 h post infection (hpi). Cultured hBMECs (10^4^ cells/well) in 96-well tissue culture plates (Nunc) were incubated in 100 μL of RPMI medium for 18 h in a humidified incubator (37 °C, 5% CO_2_) until they became confluent. Tachyzoites of *N. caninum* were added, in triplicate, to hBMECs at MOI 2 for 2 h, followed by removal of the medium and washing with fresh RPMI medium to remove extracellular, non-attached, parasites. Each well was filled with 100 μL of fresh RPMI medium and the plates were incubated at the above culture conditions. Dimethyl sulfoxide (DMSO, 0.1%) was used as a control. At the indicated time points after infection, MTT was added to each well (to a final concentration of 0.5 mg/mL), and incubation was continued for a further 3–4 h in the dark at 37 °C. The cells were then incubated for 1 h in 100 µL of solubilizing solution (10% sodium dodecyl sulfate in 0.01 M HCl). The optical density (OD) was measured using a microtiter plate reader at 570nm. The experiment was performed in triplicate.

### 4.5. Assessment of Mitochondrial Membrane Potential

The mitochondrial (Mt) membrane potential (ΔΨm) represents an important parameter for cellular metabolism in general and the Mt energy status in particular. Therefore, ΔΨm was measured in mock- and *N. caninum*-infected cells with a ΔΨm-sensitive fluorescent MitoTracker^®^ Green FM dye that labels mitochondria irrespective of oxidative activity and can be used to assess Mt mass. hBMECs were plated (10^4^ cells/well) in a black 96-well microtiter plate with a clear bottom and were infected by *N. caninum* tachyzoites at a MOI of 2. At 6, 24 and 48 hpi, cells were incubated at 37 °C with 600 nM of the MitoTracker^®^ Green FM (Molecular Probes, Eugene, OR, USA) in PBS for 30 to 45 min and washed twice with prewarmed PBS. 1uM FCCP, a mitochondrial OXPHOS uncoupler, was used as a positive control for 1 h before plate reading. The fluorescence emission of MitoTracker^®^ Green FM was analyzed using a multilabel microplate reader at 480/520 nm excitation/emission.

### 4.6. Propidium Iodide Staining and Flow Cytometry

To determine the effect of *N. caninum* on host cell cycling, propidium iodide (PI) staining and flow cytometry analysis were performed as previously described [18]. *N. caninum*-infected (MOI 2) and uninfected control hBMECs were detached using 0.1% (wt/vol) trypsin-0.2% (wt/vol) EDTA. Following centrifugation (400× *g* for 10 min), the cells were fixed and permeabilized in ice-cold 70% ethanol for 60 min. Following washing with PBS, cells were incubated with freshly prepared PI staining solution (containing 50 µg of PI, 100 kunitz units of ribonuclease A (Sigma, UK), and 1 mg glucose in 1 mL of PBS) at ambient temperature in the dark for 60 min. After staining, the cells were run through a Beckman Coulter Altra flow cytometer (Beckman Coulter). Approximately 1 × 10^4^ cells were measured for each sample using the 488nm laser for excitation and red fluorescence (>600 nm) and side scatter. After gating and removal of cell debris within the total cell population, the number of cells that were dead (apoptotic, hypo-diploid), normal (diploid, 2n), and dividing/mitotic, were recorded on a Global Worksheet.

### 4.7. RT^2^ Profiler PCR Array for Mitochondria-Related Gene Expression

Total RNAs from *N. caninum*-infected hBMECs (MOI 2) and matched (control) uninfected cells were isolated using RNeasy kit (Qiagen, Valencia, CA, USA). The extracted RNA yield and purity were determined with absorbance of 260nm and 280nm measured using the NanoDrop ND-1000 UV/Vis spectrophotometer according to manufacturer’s instructions (NanoDrop Technologies, Wilmington, DE, USA). RNA integrity was confirmed using the Agilent Bioanalyzser 2100 and RNA Nano 6000 Labchip kit, with ~250 ng RNA loaded on the chip according to the manufacturer’s instruction (Agilent Technologies, Palo Alto, CA). Reverse-transcriptase (RT) reaction was performed with 1000 ng of total RNA using an RT^2^ First Strand Kit (SABioscience). Random-primed cDNAs were processed for quantitative real-time reverse-transcriptase PCR (qRT-PCR) of 84 genes involved in biogenesis and function of mitochondria and 12 housekeeping genes including internal controls using an RT^2^ Profiler™ PCR Array Kit (RT^2^ ProfilerTM PCR Array Human Mitochondria, PAHS-087A, SABioscience) and an ABI 7300 real-time PCR system (Applied Biosystems, Foster City, CA, USA). PCR products were quantified by measuring SYBR Green fluorescent dye incorporation with ROX dye reference. Four independent pair-wise comparisons were performed to evaluate scored differences in gene changes with a correlation coefficient of *R* > 0.990. An integrated web-based software package for the PCR Array System performed ^ΔΔ*C*^t-based fold-change calculations from the uploaded raw threshold cycle data. The data were normalized and analyzed using fold change from control with positive values indicating an upregulation and negative values indicating a downregulation compared to control. Genes with >1.5 fold change in expression at a *p*-value of < 0.05 from control were considered as DEGs.

### 4.8. FTIR Spectroscopy Analysis

Cell monolayers of hBMECs were incubated at 37 °C with 5% CO_2_ in 6-well tissue culture plates. Cells were infected with *N. caninum* at MOI of 2, and after 2 h the medium including extracellular (unattached) tachyzoites was removed from each well and replaced with fresh medium. The infected cells were further incubated for 3, 24 and 48 hpi. At each time point, culture medium was discarded, and the cells were washed twice with PBS to remove any traces of the culture medium. Cells were trypsinized, centrifuged and resuspended in 4% paraformaldehyde (PFA). The fixed cell suspension was then pipetted on zinc selenide windows and left to air-dry. FTIR measurements were performed in the transmission mode using a liquid-nitrogen-cooled MCT detector fitted to an FTIR microscope (Bruker Hyperion 2000 microscope [Bruker Optics, Coventry, UK]) coupled to an FTIR spectrometer (Bruker Tensor 27 FTIR spectrometer [Bruker Optics, Coventry, UK]). The spectra were obtained in the wave number range of 4000–700 cm^−1^. Spectral resolution was set at 4 cm^−1^. The spectra collected were an average of 64 scans in order to increase the signal-to-noise ratio. Due to the possible heterogeneity of the samples caused by the different thickness of the cell layers at various regions of the prepared sample, and due to the possible presence of traces of PFA crystals, particularly in areas with a lower cell density, appropriate sample regions were selected. Background measurements were performed regularly on empty (cell-free) areas of the window to minimize noise and the signal from moisture. The sample area analyzed was 100 μm × 100 μm, to improve the signal/noise ratio. From each sample, at least 50 independent spectra were recorded. The experiment at all time points (3, 24 and 48 hpi) was performed in triplicate. The ratio analysis was performed to measure the change of chemical content in host cells with respect to different time points after infection. The raw spectra were first calibrated using cubic spline function from MATLAB 2017a. This has enabled the alignment of all the wavenumber to the same step size. All spectra were then normalized to zero mean and unity standard deviation.

### 4.9. Synchrotron-Based XRF Mapping

hBMECs were seeded on Quartz slides (UQG Ltd., Cambridge, UK) until they formed a monolayer after 24 h. Then, tachyzoites were added to the cell monolayer at a MOI of 2. Quartz slides were also seeded with cells, but without addition of parasites and were used as controls. At 3, 24 and 30 hpi, three slides with infected or non-infected control cells were washed 3X in sterile PBS and fixed in 4% PFA for 30 min. Then, washed 3X again in sterile PBS and left at 4 °C in PBS until measurements. All experiments were conducted at least in triplicate. A high-resolution synchrotron radiation X-ray fluorescence (μSR-XRF) imaging approach was used to map and quantify the levels of Zinc (Zn), Copper (Cu) and Iron (Fe) within the hBMECs. Control samples were also analyzed to establish a baseline for the levels of these metals. Controls and infected cells with incubation times of 3, 24 and 30 hpi were measured allowing the evaluation of the metal content levels within the cells at different incubation stages post infection. Imaging of the elemental maps of the three metals was performed on areas ~ 200 × 200 µm^2^ from each sample on I18 with the beam focused to 3 × 5 μm^2^ and a dwell time of 5 s per point. A thin-film reference material for XRF measurements (AXO Dresden GmbH) was used to evaluate the metal content in terms of ppms. The concentration maps were produced using Pymca [48] that allows fitting of the acquired spectra and translating the intensity maps into concentration distributions by taking into account an approximate cellular matrix composition. From the concentration maps regions of interest within cells (three regions per sample) were statistically evaluated producing average concentration values for each of the three incubation times. In an effort to extract the most appropriate information possible, regions that contained single cells in contrast to a number of overlapping cells were identified and hotspots with excessive elemental counts were excluded from the measurement. Statistical significance was evaluated using the Student’s t-test (two tailed, assuming unequal variance) in the Excel software program. A *p*-value of ≤0.05 was considered the cut-off for a significant difference.

### 4.10. Microarray Analysis

Here, in addition to studying the transcriptional response of hBMECs to infection by *N. caninum*, we also studied the transcriptome of rBMVECs to explore whether cerebromicrovascular endothelial cell response to infection varies between different host species (i.e., human versus rat).

#### 4.10.1. Infection Protocol

Approximately 48 h before the parasite culture was likely to have lysed its host Vero cells; the rBMVEC and hBMEC cultures were trypsinized. An aliquot of the culture suspension was used to count the number of cells/mL using a hemocytometer. As shown in Supplementary Figure S2, 6-well plates were seeded with rBMVECs or hBMECs at a density of approximately 6 × 10^3^ cells/cm^2^. Plates used for rBMVECs were pre-prepared with AFS. The final volume in each well was made up to 2 mL with the corresponding culture media (RBMVEC growth medium for rBMVECs and RPMI for hBMECs). The plates were then incubated at 37 °C, 5% CO_2_ for 48 hr. Before infection, the culture media were removed from the seeded plates. Three wells in each plate were infected with purified tachyzoites in 2 mL fresh culture media at a MOI of 2. Three wells were mock inoculated with 2 mL of fresh culture media and used as controls. All plates were incubated at 37 °C and 5% CO_2_ for 2 h. Then, culture media were removed from all wells, both infected and control plates, and 2 mL of fresh corresponding culture media were added. All plates were incubated at 37 °C and 5% CO_2_ for further 24 h.

#### 4.10.2. RNA Isolation and Quantification

Total RNA was isolated from three technical replicates of hBMEC- or rBMVEC-infected cultures and the corresponding controls at 24 hpi using the RNeasy^®^ Plus mini kit (QIAGEN, UK), according to the manufacturer’s instructions for “Isolation of RNA from animal cells”. Approximately 1:10 dilutions of total RNA isolations were prepared in RNase-free H_2_O and stored at –80 °C. RNA concentrations and quality were determined using a Nanodrop spectrophotometer (Thermo Fisher Scientific, Wilmington, DE, USA). Only RNAs that passed the quality control cut-offs of A260/A280 ratio of ≥1.8 and A260/A2830 ratio of ≥1.6 were used. Moreover, RNA integrity number (RIN) was determined using the Agilent Bioanalyzer RNA 6000 Nano LabChip Kit (Agilent Technologies, Santa Clara, USA) in combination with the Agilent 2100 Bioanalyzer according to the manufacturer’s instructions.

#### 4.10.3. RNA Amplification and Microarray

RNA samples from the rBMVECs (infected and control) with RIN >8 were submitted to Source Bioscience (Nottingham, UK) for hybridization to Affymetrix Rat Genome 230 2.0 array. Each microarray contained a total of 31,099 probe sets, encoding for over 28,000 rat genes. Each sample was hybridized to a separate array. RNA samples from the hBMECs were analyzed using Affymetrix GeneChip^®^ Human Gene 1.0 ST Array (Affymetrix, Santa Clara, CA, USA) by the transcriptomics service at Nottingham Arabidopsis Stock Centre (NASC). The human array consisted of approximately 33,252 probe sets covering over 28,869 genes. The process was performed following the Affymetrix instructions. Briefly, double-stranded cDNA was synthesized by a chimerical oligonucleotide with oligo-dT and T7 RNA polymerase. The amplification and labeling processes were monitored using a GeneChip^®^ Eukaryotic Poly-A RNA Control Kit (Affymetrix) with exogenous positive controls that were spiked into the total RNA before cDNA synthesis. About 25 µg of each biotinylated cRNA preparation was fragmented and placed in hybridization cocktail containing biotinylated hybridization controls (GeneChip^®^ Expression Hybridization Controls, Affymetrix). Microarray scanned images were obtained with a GeneChip Scanner 3000 7G (Affymetrix) using the default settings. Images were visually inspected to eliminate hybridization artifacts.

#### 4.10.4. Bioinformatic Analysis

Gene expression data were analyzed using Partek Genomics Suite 6.6 software (Partek Incorporated). The raw CEL files were normalized using the RMA background correction with quantile normalization, log base 2 transformation and mean probe-set summarization with adjustment for GC content. DEGs were identified by a two-way ANOVA. DEGs were considered significant if *p*-value was ≤ 0.05 and fold change of >1.5 or <−1.5. GO enrichment analysis of hBMEC data set was performed on the genes found to be over- or under-expressed in infected versus control cells using the three following ontologies: biological process, molecular function and cell compartment. GO analysis could not be done on rBMVECs because Partek software did not have the rat ontology files. Interestingly, Ensembl biomart was used to identify potential cross-species orthologues and no common genes were found. This may be attributed species differences or related to the low number of significantly DEGs identified.

#### 4.10.5. Microarray Data Accession Numbers

The microarray data produced in the present study have been submitted to the European Bioinformatics Institute (EMBL-EBI) and deposited in ArrayExpress database (https://www.ebi.ac.uk/arrayexpress/) under accession number E-MTAB-9411.

### 4.11. Statistical Analysis

Statistical analysis was performed using GraphPad Prism version 7.02 (GraphPad^®^ Software Inc., San Diego, CA, USA). Statistical significance of differences in host cell viability, mitochondrial function, cell cycle between control (uninfected) and infected cells at different times post infection were determined by two-way analysis of variance (ANOVA) and Bonferroni’s post-hoc test for multiple comparisons as appropriate. A *p* value of <0.05 was considered significant.

## 5. Conclusions

We presented our attempt to correlate the findings obtained from investigation of the viability, bioenergetic, chemical signature, elemental profile, and gene expression of BBB endothelial cells infected by *N. caninum*. The FTIR spectroscopic fingerprinting analysis of hBMECs at 3, 24 and 48 hpi showed that *N. caninum* infection caused significant changes in the chemical composition of infected cells. The biggest changes were detected early at 3 hpi, followed by less alterations at 24 hpi, and then again more significant changes were noticed at 48 hpi, correlating with parasite invasion and egress phases at early and late stages of the infection cycle, respectively. Elemental mapping using μSR-XRF demonstrated the important role of Zn in mediating host–*N. caninum* interaction. These findings demonstrate the potential of FTIR spectroscopy and μSR-XRF for determining the changes in the chemical composition and metal content of host cells during infection. We also examined the patterns of gene expression in human versus rat brain microvascular endothelial cells. Despite the functional similarity between both cell lines, their expression patterns differ in response to *N. caninum* infection, indicating species-specific differences in response to infection. Microarray analysis also enabled identification of host genes modulated during infection, allowing more understanding of genes and processes altered during infection. The effect of *N. caninum* on gene expression in endothelial cells, although minimal, influences a number of key processes, including immune response, cell signaling, lipid synthesis and cell death. Elucidation of the repertoire of host cell bioenergetics, macromolecules, trace elements, genes, and processes that are altered by *N. caninum* infection should improve understanding of the molecular events that shape *N. caninum* interaction with BBB endothelial cells.

## Figures and Tables

**Table 1:** Differentially expressed genes (DEGs) identified by the mitochondrial real-time PCR array in hBMECs at 24 h after infection.

Symbol	Gene Name ^a^	Fold Change ^b^	Mitochondrial Function
*ndufc2*	NADH dehydrogenase (ubiquinone) 1, subcomplex unknown, 2, 14.5 kDa	2.3784	Complex I
*cyc1*	Cytochrome c-1	2.3784	Complex III
*atp4a*	ATPase, H+/K+ exchanging, alpha polypeptide	2.3295	OXPHOS
*ndufa8*	NADH dehydrogenase (ubiquinone) 1 alpha subcomplex, 8, 19 kDa	1.9319	Complex I
*sdhc*	Succinate dehydrogenase complex, subunit C, integral membrane protein, 15 kDa	1.9319	Complex II
*ndufs2*	NADH dehydrogenase (ubiquinone) Fe-S protein 2, 49 kDa (NADH-coenzyme Q reductase)	1.7777	Complex I
*sdha*	Succinate dehydrogenase complex, subunit A, flavoprotein (Fp)	1.7654	Complex II
*ndufb10*	NADH dehydrogenase (ubiquinone) 1 beta subcomplex, 10, 22 kDa	1.7291	Complex I
*ndufs6*	NADH dehydrogenase (ubiquinone) Fe-S protein 6, 13 kDa (NADH-coenzyme Q reductase)	1.7053	Complex I
*rpl13a*	Ribosomal protein L13a	1.6818	
*uqcr11*	Ubiquinol-cytochrome c reductase, complex III subunit XI	1.6702	Complex III
*atp5c1*	ATP synthase, H+ transporting, mitochondrial F1 complex, gamma polypeptide 1	1.6702	Complex V
*uqcrc1*	Ubiquinol-cytochrome c reductase core protein I	1.6818	Complex III
*ppa2*	Pyrophosphatase (inorganic) 2	1.6818	Complex V
*ndufa11*	NADH dehydrogenase (ubiquinone) 1 alpha subcomplex, 11, 14.7 kDa	1.6702	Complex I
*ndufv1*	NADH dehydrogenase (ubiquinone) flavoprotein 1, 51 kDa	1.6021	Complex I
*atp6v1e2*	ATPase, H+ transporting, lysosomal 31 kDa, V1 subunit E2	1.5911	Phosphorylative mechanism
*uqcrh*	Ubiquinol-cytochrome c reductase hinge protein	1.5583	Complex III
*b2m*	Beta-2-microglobulin	1.5476	Complex I
*ndufs4*	NADH dehydrogenase (ubiquinone) Fe-S protein 4, 18kDa (NADH-coenzyme Q reductase)	1.5263	Complex I
*cox5b*	Cytochrome c oxidase subunit Vb	1.5052	Complex IV

^a^ DEGs that were deemed significant (*p*-value < 0.05) and had fold change (>1.5 or <−1.5) are shown. ^b^ Fold change represents the ratios of gene expression levels (infected relative to control cells) at 24 hpi.

**Table 2:** Top differentially expressed genes (DEGs) detected in human and rat endothelial cells after 24 h of *N. caninum* infection.

Symbol	Gene Name ^a^	Fold Change ^b^	Function
**human brain microvascular endothelial cell (hBMECs)**			
**Downregulated**			
*mtnd2*	NADH dehydrogenase 2	−4.143	Bioenergetics
*insig1*	Insulin induced gene 1	−1.705	Innate immunity
*hist1h2ak*	Histone cluster 1, H2ak	−1.678	Unknown
*hmgcs1*	3-hydroxy-3-methylglutaryl-CoA synthase 1	−1.636	Lipid synthesis
*snora7b*	Small nucleolar RNA, H/ACA box 7B	−1.600	Innate resistance
**Upregulated**			
*tob2*	Transducer of ERBB2, 2	1.860	Cell cycle
*Bivm*	Basic, immunoglobulin-like variable motif containing	1.520	Unknown
**rat brain microvascular endothelial cell (rBMVECs)**			
**Downregulated**			
*rsad2*	Radical S-adenosyl methionine domain containing protein 2	−2.046	Immune response
*cxcl2*	Chemokine (C-X-C motif) ligand 2	−2.040	Immune response
*trib3*	Tribbles homolog 3 (Drosophila)	−1.818	Fatty acid biosynthesis
*mdn1*	Midasin homolog (yeast)	−1.626	Transcription
*ifi27l2b*	Interferon, alpha-inducible protein 27 like 2B	−1.565	Immune response
*cxcl10*	Chemokine (C-X-C motif) ligand 10	−1.544	Immune response
*herc1*	HECT and RLD domain containing E3 ubiquitin protein ligase family member 1	−1.543	Membrane trafficking
*gadd45a*	Growth arrest and DNA-damage-inducible, alpha	−1.528	Apoptosis
*Isg15*	ISG15 ubiquitin-like modifier	−1.508	Immune response
**Upregulated**			
*gem*	GTP binding protein overexpressed in skeletal muscle	1.555	Signal transduction

^a^ DEGs that were deemed significant (*p*-value ≤ 0.05) and had fold change (>1.5 or <−1.5) are shown.; ^b^ Fold change represents the ratios of gene expression levels (infected relative to control cells) at 24 hpi.

## Supplementary Materials

The following are available online at https://www.mdpi.com/2076-0817/9/9/710/s1, Supplementary Figure S1: The average spectra of control and infected hBMECs at 3, 24 and 48 hpi. The dotted lines indicate the +/− one standard deviation of the averaged absorbance value, Supplementary Figure S2: Schematic of the experimental design of gene expression profiling of human brain microvascular endothelial cells (hBMECs) and rat brain microvascular endothelial cells (rBMVECs) under *Neospora caninum* attack. *N. caninum* tachyzoites of Liverpool strain were added to monolayers of each cell line grown in 6-well culture plates in 3 replicates, for 24 h. Three wells were infected by tachyzoites and three parallel wells were mock-infected using culture medium only. RNAs were isolated from cells from each well after 24 h of infection and were processed for microarray analysis as described in the materials and methods.
