# Supplementary figures and images for: Impact of Neospora caninum Infection on the Bioenergetics and Transcriptome of Cerebrovascular Endothelial Cells
Source: Pathogens. 2020 Aug 28;9(9):710. doi: 10.3390/pathogens9090710 (PMC7559149; doi:10.3390/pathogens9090710)

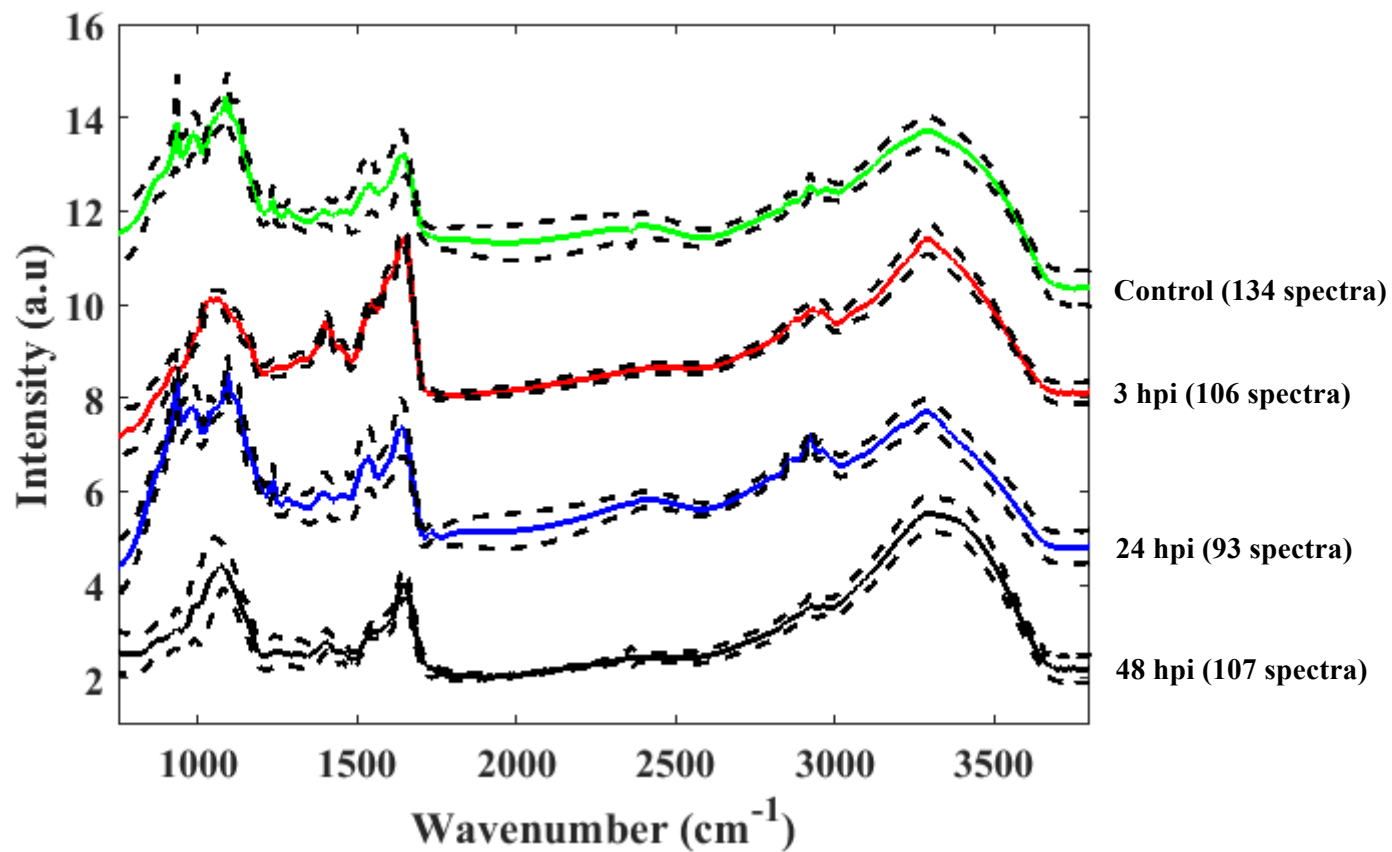

Supplement: Supplementary file 1 [file pathogens-09-00710-s001.pdf]
